# Supplementary material for: No Excess Mortality up to 10 Years in Early Stages of Breast Cancer in Women Adherent to Oral Endocrine Therapy: A Probabilistic Graphical Modeling Approach
Source: Int J Environ Res Public Health. 2022 Mar 18;19(6):3605. doi: 10.3390/ijerph19063605 (PMC8950380; doi:10.3390/ijerph19063605)
Supplement: Supplementary file 1 [file ijerph-19-03605-s001.zip › ijerph-1622644-supplementary.pdf]

Supplementary material for: “No excess mortality up to 10 years in early stages of breast cancer in women adherent to oral endocrine therapy: a probabilistic graphical modeling approach”

by Ramon Clèries et al 2022

1.- R Code to run simulations and ComSynSurData ….. page 2

2.- WinBUGS model…………………………………… ....page 8

1.- R Code to run simulations and ComSynSurData

```
# Functions need

gen.synthetic.data.K<-function(data.TMP,K.t,N.Sim=10000000)
{
cad.dmod<-dmod( ~.^.,data=data.TMP)
mnew2<-stepwise(cad.dmod, k=K.t)
plot(mnew2)
ug2.dmod <- ugList( terms( mnew2 ) )
cad.gin.dmod2 <- compile( grain( ug2.dmod, data=data.TMP, smooth=0.1 ));
simulate(cad.gin.dmod2,n=N.Sim)
}

gen.synthetic.data.TEST<-function(data.TMP,N.Sim=10000000)
{
mod.dmod<-dmod( ~.^.,data=data.TMP)
mnew2<-stepwise(mod.dmod, criterion="test")
plot(mnew2)
ug2.dmod <- ugList( terms( mnew2 ) )
mod.gin.dmod2 <- compile( grain( ug2.dmod, data=data.TMP, smooth=0.1 ));
simulate(mod.gin.dmod2,n=N.Sim)
}

IBrier.Surv<-function(data.sy.t,data.BASE.t)
{
smod.tmp <- Surv(data.BASE.t$Followup, data.BASE.t$Exitus)
KM.tmp <- survfit(Surv(Followup, Exitus) ~ 1,data=data.sy.t)
# integrated Brier score up to max(time)
sbrier(smod.tmp, KM.tmp)[[1]]
}

ComSynSurData<-function(df.base,list.df.sim,age.low,age.up)
{
```

```
# df.base: "real" data base, observed data

# list.df.sim: list of simulated data bases generated, that is "synthetic"
datasets

# age.low: lower limit for each each age-group considered

# age.up: upper limit for each age-group considered

# There must be an "Strata" variable in df.base (for instance, Stage at
diagnoses, with classes "I","II" and "III")




if (c("Strata","Age","Exitus","Followup")%in%names(df.base))

{

# Define Strata levels

L.Strata<-sort(unique(df.base$Strata))

N.Strata<-length(L.Strata)

#Define age levels

N.Ages<-length(age.low)


# Number of Simulated Datasets provided by the user

N.df.sim<-length(list.df.sim)


# Initialize the list of L Synthetic Datasets

list.db.L<-list()

list.syn.L<-list()


# for each Strata, Initialize the matrix of Age-groups X N.df.sim


mat.Brier.tmp<-matrix(0,N.Ages,N.df.sim)

list.mat.Brier.tmp<-list()


#### Iterative process for generating Brier Scores


### Selecting the "best" subset according to the minimum Score for each age-
group


df.AG<-as.data.frame(list.df.sim[[1]][1,])
```

```
for (i.strata in 1:N.Strata)

{

for (i.age in 1:N.Ages)

{

# Select Strata of data base

df.base.tmp<-df.base[df.base$Age>age.low[i.age] & df.base$Age<=age.up[i.age] &
df.base$Strata==L.Strata[i.strata],]

print(summary(df.base.tmp))

# Selet Strata for each simulated dataset

list.df.sim.tmp<-list()

for (i.df.sim in 1:N.df.sim)

{

df.sim.tmp<-list.df.sim[[i.df.sim]]

df.sim.tmp<-df.sim.tmp[df.sim.tmp$Age>age.low[i.age] &
df.sim.tmp$Age<=age.up[i.age] & df.sim.tmp$Strata==L.Strata[i.strata],]


list.df.sim.tmp[[i.df.sim]]<-df.sim.tmp


Ibrier.score.tmp<-IBrier.Surv(df.sim.tmp,df.base.tmp)

mat.Brier.tmp[i.age,i.df.sim]<-Ibrier.score.tmp

}


min.Brier<-which.min(mat.Brier.tmp[i.age,])

df.AG<-as.data.frame(rbind(df.AG,list.df.sim.tmp[[min.Brier]]))


list.mat.Brier.tmp[[i.strata]]<-as.data.frame(round(mat.Brier.tmp,6))

names(list.mat.Brier.tmp[[i.strata]])<-paste("Syn",c(1:N.df.sim),sep="")

row.names(list.mat.Brier.tmp[[i.strata]])<-paste("AgeGoup",c(1:N.Ages),sep="")

}

# Re initialize matrix

mat.Brier.tmp<-matrix(0,N.Ages,N.df.sim)

}

return.tmp<-list(list.mat.Brier.tmp,df.AG)

}

else
```

```
{

print("Error: Please include Strata, Age, Exitus, Followup as numerical
variable in your database")

return.tmp<-NULL

}

return.tmp

}



# R function to be used for comparing survival in our paper, it compares
survival according to Stage.

# The user must change STage by any other variable considered in his/her own
study.

compare.surv<-
function(data.base.TMP,data.sim.TMP,age.low=c(0,49,59),age.up=c(49,59,74))

{

L.STAGES<-levels(data.base.TMP$STAGE)

for (i.Strata in 1:length(L.STAGES))

{

for (i.age in 1:length(age.low))

{

data.base.TMP.age<-data.base.TMP[data.base.TMP$STAGE==L.STAGES[i.Strata] &
data.base.TMP$AGE>age.low[i.age] & data.base.TMP$AGE<=age.up[i.age],]

data.sim.TMP.age<-data.sim.TMP[data.sim.TMP$STAGE==L.STAGES[i.Strata] &
data.sim.TMP$AGE>age.low[i.age] & data.sim.TMP$AGE<=age.up[i.age],]

surv.base<-survfit(Surv(FU,EXITUS)~1,data=data.base.TMP.age)

surv.sim<-survfit(Surv(FU,EXITUS)~1,data=data.sim.TMP.age)

plot(surv.base,xlab="Years",ylab="Observed Survival")

lines(surv.sim$surv,col="red")

title.paste<-paste("Stage:",L.STAGES[i.Strata],"
Age:(",age.low[i.age],",",age.up[i.age],"]")

title(title.paste)

}

}

}



#### Generating Graphical Model and simulating from it



# 1.- Load R packages (libraries)
```

```
library(cuRe)

library(survival)

library(relsurv)

library(R2WinBUGS)

library(survexp.fr)

library(readxl)

library(arsenal)

library(survival)

library(survminer)

library(ggfortify)



library(neuralnet)

library(MASS)

library(nnet)

library(ggplot2)

library(caret)

library(gRain)

library(gRim)

library(Rgraphviz)

library(survival)

library(psych)

library(grid)

library(gridExtra)


library(relsurv)

library(rms)

library(ipred)
```

```
# 2.- Load Data Base

# data.file includes the original data base with no missing information.

DATA.BASE<-read.table(data.file)


#### GEN SYNTHETIC DATA

set.seed(100)

DATA.K1<-gen.numerical.and.rep.vars(gen.synthetic.data.K(DATA.BASE,1,1000000))

x11()

DATA.AIC<-
gen.numerical.and.rep.vars(gen.synthetic.data.K(DATA.BASE,2,1000000))

x11()

DATA.BIC<-
gen.numerical.and.rep.vars(gen.synthetic.data.K(DATA.BASE,log(dim(DATA.AN.AD.N
AD.BASE)[1])-1,1000000))

x11()

DATA.TEST<-
gen.numerical.and.rep.vars(gen.synthetic.data.TEST(DATA.BASE,1000000))

x11()


# 3.- Generate repeated variables, in order to apply ComSynSurData

DATA.BASE.REP<-gen.rep.vars(DATA.AN.AD.NAD.BASE)


# 4.- Apply ComSynSurData


list.mat.SEL.SIM<-
ComSynSurData(df.base=DATA.BASE.REP,list.df.sim=list(DATA.K1,DATA.AIC,DATA.BIC
,DATA.TEST),age.low=c(0,49,59),age.up=c(49,59,74))


# 5.- Get Brier Scores Table


Table.Brier.Scores<-list.mat.SEL.SIM[[1]]


# 6.- Get the "Combined" synthetic dataset


Comb.Surv<-list.mat.SEL.SIM[[2]]
```

2.- WinBUGS model

Requires the following data

N: Number of years

Y: person-years at risk (sum of follow-up times of all patients at-risk of death in one year)

E: expected number of deaths (annual); # O: observed number of deaths (annual);

```
model{

log(mu[1])<-log(Y[1])+delta[1]

O[1]~dpois(mu[1])

delta[1]~dnorm(0,0.001)

lambda.O[1]<-mu[1]/Y[1]

lambda.E[1]<-E[1]/Y[1]

diff.X[1]<-lambda.O[1]-lambda.E[1]

diff.X2[1]<-pow(diff.X[1],2)

lambda.X[1]<-pow(diff.X2[1],0.5)


for (i.t in 2:N)

{

log(mu[i.t])<-log(Y[i.t])+delta[i.t]

O[i.t]~dpois(mu[i.t])

delta[i.t]~dnorm(delta[(i.t-1)],0.001)

lambda.O[i.t]<-mu[i.t]/Y[i.t]

lambda.E[i.t]<-E[i.t]/Y[i.t]

diff.X[i.t]<-lambda.O[i.t]-lambda.E[i.t]

diff.X2[i.t]<-pow(diff.X[i.t],2)

lambda.X[i.t]<-pow(diff.X2[i.t],0.5)

}


# PCa:crude probability of death due to cancer

# PCo:crude probability of death due to other causes


OS[1]<-exp(-mu[1]/Y[1])

ES[1]<-exp(-E[1]/Y[1])

RS[1]<-OS[1]/ES[1]
```

```
PCa[1]<-ES[1]*RS[1]*lambda.X[1]
POC[1]<-1-PCa[1]-OS[1]

for (i.t1 in 2:N)
{
OS[i.t1]<-OS[i.t1-1]*exp(-mu[i.t1]/Y[i.t1])
ES[i.t1]<-ES[i.t1-1]*exp(-E[i.t1]/Y[i.t1])
RS[i.t1]<-OS[i.t1]/ES[i.t1]
PCa[i.t1]<-PCa[(i.t1-1)]+ES[i.t1]*RS[i.t1]*lambda.X[i.t1]
POC[i.t1]<-1-PCa[i.t1]-OS[i.t1]
}

# CRS5:conditional survival
CRS5[1]<-RS[6]/RS[1]
ER5[1]<-1-CRS5[1]
for (i.t2 in 2:5)
{
CRS5[i.t2]<-RS[6+(i.t2-1)]/RS[(i.t2)]
ER5[i.t2]<-1-CRS5[i.t2]
}

}
```
